# Phenolic acid reduction in *Fritillaria taipaiensis* rhizosphere via organic fertilization

**DOI:** 10.1080/15592324.2025.2554917

**Published:** 2025-09-03

**Authors:** Wenwu Yang, Jinjin Li, You Zhou, Yuhan Wang, Wenting Wenting, Nong Zhou, Qiang-Sheng Wu

**Affiliations:** a Chongqing Institute for Food and Drug Control, Chongqing, China; b Chongqing Key Laboratory of Development and Utilization of Genuine Medicinal Materials in Three Gorges Reservoir Area, Chongqing, China; c College of Biology and Food Engineering, Chongqing Three Gorges University, Chongqing, China; d College of Pharmacy, Chongqing Three Gorges Medical College, Chongqing, China; e Hubei Key Laboratory of Spices & Horticultural Plant Germplasm Innovation & Utilization, College of Horticulture and Gardening, Yangtze University, Jingzhou, China

**Keywords:** Fertilization, *Fritillaria taipaiensis*, long-term monoculture, phenolic acid accumulation, rhizosphere

## Abstract

*Fritillaria taipaiensis* is a valuable traditional Chinese medicinal plant that is prone to germplasm degradation during long-term continuous monoculture. Allelopathic autotoxicity, which is mediated primarily by phenolic acids, is considered a major factor contributing to this degradation. To reveal the accumulation patterns of phenolic acids in the rhizospheric soil of *F. taipaiensis* under continuous monoculture, five phenolic acids (*p*-hydroxybenzoic acid, vanillic acid, syringic acid, *p*-coumaric acid, and ferulic acid) in the rhizospheric soil of *F. taipaiensis* across 1−5 y, and various fertilizer regimes (chemical fertilizer, chemical fertilizer + organic fertilizer, and organic fertilizer) were determined to assess their accumulation characteristics, along with soil fertility parameters. The result showed that the levels of available nitrogen, Olsen-phosphorus, and available potassium in chemical fertilizer and chemical fertilizer + organic fertilizer, along with the organic matter content in all three soil samples, showed a decreasing trend over time, while organic fertilizer exhibited significant fluctuations without a clear pattern. The phenolic acid content in the rhizospheric soil initially increased and then generally decreased in later stages. After 5 y of cultivation, the soils treated with organic fertilizer exhibited lower phenolic acid levels than those treated with chemical fertilizer. The accumulation patterns of individual phenolic acids varied with fertilizer type and cultivation period, with organic fertilizer showing the most consistent patterns across all phenolic acids. There was a positive correlation among the five phenolic acids, along with a significant positive correlation between soil organic matter and vanillic acid and ferulic acid. These findings suggest that long-term monoculture leads to distinct accumulation characteristics of phenolic acids in the rhizospheric soil of *F. taipaiensis*, and the application of organic fertilizer can mitigate such accumulation.

## Introduction

*Fritillaria taipaiensis* is a perennial herb belonging to the Liliaceae family. Its bulbs contain a variety of active ingredients, including alkaloids, steroids, purines, and fatty acids, which are known for their ability to reduce body temperature, alleviate cough and asthma, and resolve phlegm.^1^ In the 2010 edition of the Pharmacopoeia of the People's Republic of China,^2^ *F. taipaiensis* has been categorized as a medical material source of Chuanbeimu, a valuable traditional Chinese medicine. In recent years, its cultivation has expanded in suitable regions.^3^ However, prolonged monoculture of *F*. *taipaiensis* for 3−4 y often leads to significant challenges, including reduced growth vigor,^4^ disease accumulation,^5^ and declining medicinal quality,^6^ severely impacting its production and quality.

The primary causes of continuous cropping obstacles and germplasm degradation under long-term monoculture are soil nutrient deficiency and plant allelopathic autotoxicity.^7^^,^^8^ The nutrients that are significantly depleted in the cultivation soil and greatly affect plant growth are key mineral elements such as nitrogen (N), phosphorus (P), potassium (K), calcium (Ca), and manganese (Mn).^9^ Phenolic acids released through root exudation, litter leaching, and plant residue decomposition are major contributors to allelopathic autotoxicity.^10^ This phenomenon has been documented in crops such as tobacco, soybean, and *Rehmannia glutinosa*, with rhizomatous medicinal plants being particularly susceptible to allelopathic autotoxicity.^11^ Soil phenolic acids, including phydroxybenzoic acid, vanillic acid, syringic acid, *p*-coumaric acid, and ferulic acid,^12^ exacerbate soil-borne diseases by altering microbial communities and promoting pathogen proliferation.^13^ Current research on *F*. *taipaiensis* under continuous cropping and long-term monoculture has focused on agronomic interventions, such as optimized water-fertilizer management, planting techniques, and disease control.^14^ However, there is a lack of reported studies on the content and change patterns of phenolic acids in the rhizospheric soil of *F*. *taipaiensis*.

To address this gap, we quantified five phenolic acids in *F*. *taipaiensis* rhizospheric soil across different cultivation durations (1–5 y) and fertilization regimes (chemical, chemical + organic, and organic fertilizers) using Ultra-High-Performance Liquid Chromatography (UPLC). Concurrently, soil properties—including available N, P, K, organic matter content, and pH—were analyzed to elucidate their relationship with phenolic acid accumulation. This study aimed to uncover the temporal patterns of phenolic acid buildup under monoculture and provide a scientific basis for sustainable *F*. *taipaiensis* cultivation.

## Materials and methods

### Site description

The sampling site is a planting base (31°23′56.11″ N, 109°50′29.93″ E-31°35′26.57″ N, 109°00′11.96″ E; 2274–2290 m above sea level) located in Lanying village, Wuxi County, Chongqing, China, with an annual average temperature of 7.2°C and the annual average precipitation of 1100–1300 mm. The soil is classified as mountain yellow-brown soil (fine-loamy, mixed, mesic Aridic Haplustalf).^13^ Prior to cultivation, the fields were uncultivated natural wasteland with uniform soil baseline conditions.

Three distinct fertilizer treatments were applied: (i) S1: chemical fertilizer (N–P–K, 15:15:15; Stanley Agricultural Group Co., Ltd, Linyi, Shandong, China) applied at a rate of 225 kg/hm^2^ each year; (ii) S2: commercial organic fertilize (Stanley Agricultural Group Co., Ltd, Linyi, Shandong, China) at a rate of 400 kg/hm^2^ each year; (iii) S3: A combination of chemical fertilizer (N–P–K, 15:15:15; Stanley Agricultural Group Co., Ltd, Linyi, Shandong, China) at 110 kg/hm^2^ and commercial organic fertilize (Stanley Agricultural Group Co., Ltd, Linyi, Shandong, China) at 200 kg/hm^2^ each year.

The field dimensions for *F. taipaiensis* were 20 × 20 m for each cultivation year, with the following designations: Y1 (1 y of cultivation), Y2 (2 y of cultivation), Y3 (3 y of cultivation), Y4 (4 y of cultivation), and Y5 (5 y of cultivation).

### Sample collection

In May 2017, rhizospheric soil samples of *F. taipaiensis* were collected from fields subjected to S1, S2, and S3, across cultivation durations of 1–5 y (Y1–Y5). For each combination of cultivation year (Y1–Y5) and fertilizer treatment (S1–S3), rhizospheric soil samples were collected from three replicates.

 The soil samples were collected from a depth of 6–10 cm below the ground using the root-shaking method.^13^ Prior to collection, any surface litter and residual cover were carefully removed. The collected soil samples were immediately placed into sterile bags, stored in an ice box, transported to the laboratory, and subsequently preserved at −80°C in an ultra-low temperature freezer for subsequent analysis.

### Determination of soil chemical variables

The soil pH value was determined using a calibrated pH meter. The soil organic matter content was quantified via the potassium dichromate oxidation‒external heating method.^15^ The soil's available N level was determined by the alkaline hydrolysis diffusion method.^14^ The soil's Olsen-P level was assessed using the molybdenum–antimony anticolorimetric method.^15^ The soil's available K level was measured using 1 mol/L ammonium acetate extraction followed by atomic absorption spectrometry.^15^

### Extraction and determination of soil phenolic acids

A 5.0 g soil sample, which had been sieved through a 100-mesh sieve, was transferred to a 50 mL centrifuge tube, followed by the addition of 25 mL of 2 mol/L NaOH. The mixture underwent ultrasonic oscillation for 30 min, followed by horizontal shaking at 190 r/min at 28°C for 12 h. After extraction, the sample was allowed to settle at room temperature for 2 h, then centrifuged at 4000 × *g*/min for 10 min. The supernatant pH was adjusted to 2.5 using 12 mol/L HCl to precipitate humic acid, and the solution was filtered. The filtrate was subjected to three sequential ethyl acetate extractions; the pooled organic phase was evaporated to dryness at 45°C under reduced pressure using a rotary evaporator. The residue was reconstituted in methanol, diluted to 5 mL in a brown volumetric flask, and filtered through a 0.22-μm millipore filter.

Following sample preparation, UPLC analysis was performed. Prior to analysis, reference standards were prepared and validated. Five phenolic acids, including *p*-hydroxybenzoic acid (DST170506-4), vanillic acid (DST180516-8), syringic acid (DST7180424-7), *p*-coumaric acid (DST171206-7), and ferulic acid (110773-2), were provided by Chengdu Desite Biotechnology Co., Ltd. (Chengdu, Sichuan, China). Each standard was dried to constant weight under reduced pressure, dissolved in pure methanol, and prepared as stock solutions at concentrations of 1.028, 0.492, 1.078, 0.334, and 0.970 mg/mL, respectively.

The chromatographic conditions tested were as follows: ACQUITY UPLC H-Class UPLC (Waters Technology (Shanghai) Co., Ltd, Shanghai, China); chromatographic column, ACQUITY UPLC BEH C18 (2.1 × 50 mm, 1.7 μm); mobile phase A, 0.1% acetic acid solution; mobile phase B, methanol; flow rate, 0.3 mL/min; sample volume, 20 µL; and column temperature was 35°C. The elution gradient was as follows: 0 min, mobile phase A 95%, mobile phase B 5%; 4 min, mobile phase A 80%, mobile phase B 20%; and 12 min, mobile phase A 70%, mobile phase B 30%.

### Data analysis

Using Microsoft Excel 2019 for one-way ANOVA variance analysis (*α* = 0.05), using SPSS version 22.0 (SPSS Inc., Armonk, NY, USA) for curve estimation and correlation analysis. The linear relationship between pairs of variables was quantified using Pearson's correlation coefficient (*R*) in SPSS software with the “Bivariate Correlation” function.

## Results

### Changes in soil nutrient levels

The levels of available N, Olsen-P, and available K in S1 and S3, as well as the organic matter content in all three soil samples (S1, S2, and S3), exhibited a declining trend with extended cultivation periods (Table 1). In contrast, the levels of available N, Olsen-P, and available K in S2 showed substantial variability over time, without a discernible pattern. The average levels of available nutrients in the rhizospheric soil of *F*. *taipaiensis* across all three fertilizer treatments were ranked as follows: S1 (155.200 mg/kg for available N, 99.260 mg/kg for Olsen-P, and 363.612 mg/kg for available K) > S3 (103.574 mg/kg for available N, 84.630 mg/kg for Olsen-P, and 326.337 mg/kg for available K) > S2 (92.983 mg/kg for available N, 36.255 mg/kg for Olsen-P, and 251.672 mg/kg for available K). The ranking for average organic matter content was: S2 (52.279  mg/g) > S3 (36.282 mg/g) > S1 (26.747 mg/g). Furthermore, the pH value increased with cultivation time across all three fertilizer treatments. Notably, the average pH value of S1 (5.58) was significantly lower than that of S2 (6.75) and S3 (6.37).

**Table 1.** Changes in soil nutrient levels of *Fritillaria taipaiensis* after three fertilizations.

	Cultivation year	Available N (mg/kg)	Olsen-P (mg/kg)	Available K (mg/kg)	Organic matter (mg/g)	pH
S1	Y1	177.477 ± 0.060a	162.167 ± 0.065a	345.875 ± 0.021e	36.038 ± 0.046c	5.04 ± 0.01h
	Y2	164.148 ± 0.020b	138.150 ± 0.028c	333.200 ± 0.023f	28.825 ± 0.073g	5.88 ± 0.01f
	Y3	163.159 ± 0.072b	124.730 ± 0.082d	477.581 ± 0.015a	26.124 ± 0.072h	5.51 ± 0.02g
	Y4	158.561 ± 0.073c	35.763 ± 0.052j	346.131 ± 0.015de	21.423 ± 0.078i	5.64 ± 0.01fg
	Y5	112.678 ± 0.032fg	35.490 ± 0.049j	315.275 ± 0.012h	21.323 ± 0.098i	5.82 ± 0.02f
S2	Y1	110.250 ± 0.052h	10.275 ± 0.043m	404.853 ± 0.008c	116.395 ± 0.021a	6.43 ± 0.03d
	Y2	147.000 ± 0.043d	54.300 ± 0.015g	173.790 ± 0.052m	44.290 ± 0.012b	6.50 ± 0.08c
	Y3	84.000 ± 0.015i	63.563 ± 0.032f	209.447 ± 0.043l	36.120 ± 0.016c	6.52 ± 0.05c
	Y4	40.833 ± 0.023m	21.675 ± 0.012l	168.293 ± 0.019n	33.536 ± 0.022d	7.47 ± 0.08a
	Y5	82.833 ± 0.036j	31.463 ± 0.010k	301.979 ± 0.052i	31.054 ± 0.020f	6.85 ± 0.04b
S3	Y1	112.000 ± 0.027g	149.466 ± 0.020b	323.882 ± 0.010g	43.574 ± 0.038b	6.21 ± 0.01f
	Y2	134.304 ± 0.033e	95.478 ± 0.009e	347.560 ± 0.010d	38.694 ± 0.084c	6.38 ± 0.05de
	Y3	113.044 ± 0.020f	96.079 ± 0.023e	412.928 ± 0.037b	33.225 ± 0.098d	6.34 ± 0.01e
	Y4	80.312 ± 0.176k	43.716 ± 0.040h	284.671 ± 0.021j	33.918 ± 0.016d	6.51 ± 0.04c
	Y5	78.212 ± 0.067l	38.413 ± 0.039i	262.643 ± 0.013k	32.001 ± 0.033e	6.39 ± 0.04de

Note: S1, chemical fertilizer application; S2, organic fertilizer application; S3, mixed application of chemical and organic fertilizer; Y1–Y5: cultivation year from 1 to 5. Different letters following the data (mean ± standard deviation, *n* = 3) indicate significant (*P* < 0.05) differences between the treatments.

### Evaluation of soil phenolic acid determination

**Figure 1.**
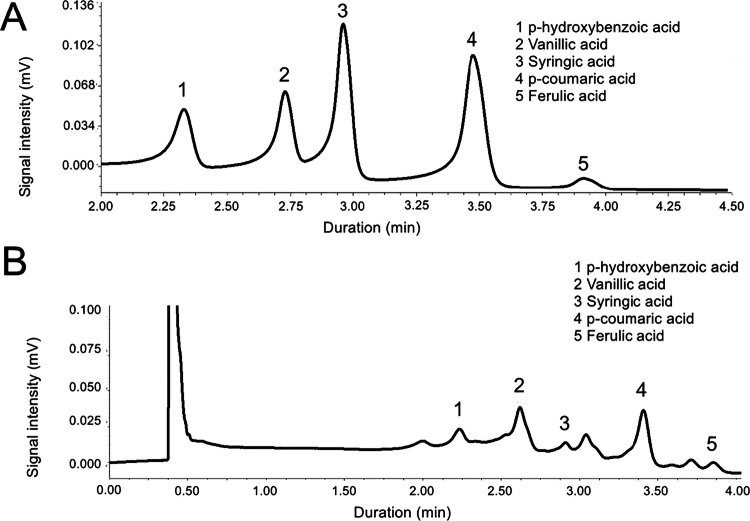
UPLC spectra of mixed reference (A) and sample (B).

In UPLC analysis, the chromatogram of the sample and standard reference are shown in Figure 1A,B. Standard curves for the five phenolic acids were established (Table 2), exhibiting strong linearity (*R*^2^* *> 0.999) and confirming the reliability of the results. The relative standard deviations (RSDs) of the chromatographic peak areas for *p*-hydroxybenzoic acid, vanillic acid, syringic acid, *p*-coumaric acid, and ferulic acid in the repetitive test were 2.64%, 3.07%, 0.58%, 1.03% and 1.02%, respectively, indicating excellent repeatability. In the stability test, the RSDs of the chromatographic peak areas for the same compounds over a 24-h period were 2.07%, 2.21%, 2.12%, 2.33%, and 1.36%, respectively, indicating good stability of the determination within this timeframe.

**Table 2.** Standard curves for the five phenolic acids in UPLC.

Phenolic acid	*R* ^2^	Regression equation	Linear range (μg/mL)
*p*-Hydroxybenzoic acid	0.9994	*y* = 13843750*x* + 7780	0.26–20.52
Vanillic acid	0.9992	*y* = 1108410*x* + 16886	2.46–196.81
Syringic acid	0.9993	*y* = 20022019*x* + 16568	0.03–2.16
*p*-Coumaric acid	0.9993	*y* = 6047085*x* + 29186	1.67–133.6
Ferulic acid	0.9991	*y* = 2637906*x* + 8690	0.24–19.43

The method demonstrated excellent recycling rates for the five phenolic acids, with average values ranging from 99.94% to 103.90% and RSDs between 2.04% and 3.09% (Table 3), confirming its high accuracy, precision, and reliability. These results validate the method's suitability for quantifying phenolic acids in *F. taipaiensis* rhizospheric soil, ensuring robust analytical performance.

**Table 3:** Recycling rates for the five phenolic acids in UPLC (*n* = 6).

Phenolic acids	Sample weight (g)	Content (μg/g)	Dosage (μg/g)	Measured quantity (μg/g)	Recycling rate	Mean	RSD
*p*-Hydroxybenzoic acid	2.5001	3.6415	3.8036	7.4414	99.90%	103.90%	2.11%
	2.5003	3.6662	3.8036	7.6570	104.92%		
	2.5001	3.7271	3.8036	7.7536	105.86%		
	2.5002	3.8015	3.8036	7.7953	105.00%		
	2.5001	3.7044	3.8036	7.6917	102.87%		
	2.5002	3.6266	3.8036	7.5395	102.40%		
Vanillic acid	2.5001	143.4237	144.6480	290.0926	101.40%	99.94%	2.18%
	2.5003	144.3227	144.6480	288.7762	99.87%		
	2.5001	143.6584	144.6480	284.1346	97.12%		
	2.5002	142.7945	144.6480	288.9407	101.04%		
	2.5001	143.9827	144.6480	292.3892	102.60%		
	2.5002	144.2066	144.6480	285.4027	97.61%		
Syringic acid	2.5001	3.9390	3.9886	8.1094	104.56%	103.57%	2.53%
	2.5003	3.9782	3.9886	8.2226	106.41%		
	2.5001	4.0440	3.9886	8.2675	105.89%		
	2.5002	3.8747	3.9886	7.8739	100.26%		
	2.5001	3.9318	3.9886	7.9442	100.60%		
	2.5002	3.9685	3.9886	8.1043	103.69%		
*p*-Coumaric acid	2.5001	50.8465	48.4300	102.1145	105.86%	102.24%	2.04%
	2.5003	50.4408	48.4300	100.0171	102.37%		
	2.5001	49.4956	48.4300	97.6824	99.50%		
	2.5002	49.1218	48.4300	98.7745	102.52%		
	2.5001	49.1206	48.4300	98.1626	101.26%		
	2.5002	49.3362	48.4300	98.6901	101.91%		
Ferulic acid	2.5001	6.0913	6.2080	12.2766	99.63%	101.92%	3.09%
	2.5003	5.6196	6.2080	12.1791	105.66%		
	2.5001	6.3350	6.2080	12.4509	98.52%		
	2.5002	6.6259	6.2080	13.0508	103.49%		
	2.5001	6.3493	6.2080	12.8670	104.99%		
	2.5002	6.1612	6.2080	12.3223	99.25%		

### Changes in soil phenolic acid levels with different cultivation years

Phenolic acid concentrations in *F*. *taipaiensis* rhizospheric soil exhibited marked variability across fertilization treatments and cultivation durations (Table 4). Vanillic acid showed the highest concentrations (3.20–268.59 μg/g), followed by *p*-coumaric acid (8.88–67.23 μg/g), while syringic acid remained the least abundant (0.06–2.36 μg/g). The ranking of the average *p*-hydroxybenzoic acid level was S3 (3.99 μg/g) > S2 (2.66 μg/g) > S1 (2.25 μg/g); the ranking of vanillic acid level was S2 (93.85 μg/g) > S1 (88.24 μg/g) > S3 (69.94 μg/g); the ranking of syringic acid level was S2 (0.80 μg/g) > S1 (0.62 μg/g) > S3 (0.43 μg/g); the ranking of *p*-coumaric acid level was S2 (37.10 μg/g) > S3 (29.02 μg/g) > S1 (27.12 μg/g); and the ranking of ferulic acid level was S2 (10.27 μg/g) > S3 (6.93 μg/g) > S1 (2.78 μg/g). Notably, the soils receiving organic fertilization (S2) consistently exhibited the highest average concentrations of vanillic acid, syringic acid, *p*-coumaric acid, and ferulic acid, underscoring the treatment's influence on phenolic acid accumulation.

**Table 4.** Changes in soil phenolic acid contents of *F. taipaiensis* after three fertilizations and cultivation years.

	Cultivation Year	*p*-Hydroxybenzoic acid (μg/g)	Vanillic acid (μg/g)	Syringic acid (μg/g)	*p*-Coumaric acid (μg/g)	Ferulic acid (μg/g)
S1	Y1	1.182 5 ± 0.0454i	39.618 9 ± 0.0402l	0.109 1 ± 0.0984h	8.878 2 ± 0.1255m	0.628 7 ± 0.0521k
	Y2	1.461 0 ± 0.0740h	64.581 7 ± 0.1260i	0.282 6 ± 0.0888fg	13.520 6 ± 0.0671j	5.115 0 ± 0.1451f
	Y3	1.843 6 ± 0.0177g	65.942 2 ± 0.2083h	0.258 9 ± 0.0095g	22.045 9 ± 0.0823h	4.341 1 ± 0.3293g
	Y4	2.226 4 ± 0.0514f	91.410 2 ± 0.0515e	1.584 1 ± 0.0233c	23.906 2 ± 0.0330g	2.213 8 ± 0.0443i
	Y5	4.489 9 ± 01276d	179.658 5 ± 0.0126b	0.852 3 ± 0.2131d	67.231 7 ± 0.0871a	1.583 2 ± 0.5704j
S2	Y1	5.090 9 ± 0.2293b	268.586 5 ± 0.0092a	1.139 0 ± 0.2971b	55.095 8 ± 0.0498b	14.016 2 ± 0.1737b
	Y2	5.388 1 ± 0.0562b	123.389 1 ± 0.0612c	2.362 0 ± 0.0891a	55.309 7 ± 0.0203b	20.649 6 ± 0.1059a
	Y3	1.802 0 ± 0.0106g	55.472 0 ± 0.1401k	0.328 0 ± 0.0719f	51.457 1 ± 0.0224c	11.710 7 ± 0.6802c
	Y4	0.790 1 ± 0.0106j	18.615 9 ± 0.2274n	0.116 6 ± 0.0754h	12.558 5 ± 0.0253k	2.896 9 ± 0.1717h
	Y5	0.213 1 ± 0.0509k	3.197 3 ± 0.1926o	0.057 1 ± 0.0063i	11.075 1 ± 0.0615l	2.073 0 ± 0.1245ij
S3	Y1	2.379 3 ± 0.2311f	61.153 7 ± 0.1515j	0.125 7 ± 0.0319h	49.899 3 ± 0.0873d	5.209 4 ± 0.0916f
	Y2	2.636 4 ± 0.0354e	83.402 1 ± 0.1437g	0.416 5 ± 0.0071f	27.067 8 ± 0.0093e	5.493 2 ± 0.1176f
	Y3	4.894 3 ± 0.0050c	86.845 7 ± 0.0407f	0.664 6 ± 0.0848e	25.613 5 ± 0.0208f	6.900 2 ± 0.1634e
	Y4	7.309 4 ± 0.0936a	93.228 7 ± 0.0015d	0.849 9 ± 0.0240d	25.505 0 ± 0.0071f	9.878 8 ± 0.0159d
	Y5	2.728 6 ± 0.2323e	25.075 6 ± 0.2306m	0.101 3 ± 0.0462h	17.025 4 ± 0.1058i	7.147 0 ± 0.1719e

Note: S1, chemical fertilizer application; S2, organic fertilizer application; S3, mixed application of chemical and organic fertilizer; Y1–Y5: cultivation year from 1 to 5. Different letters following the data (mean ± standard deviation, *n* = 3) indicate significant (*P* < 0.05) differences between the treatments.

**Figure 2.**
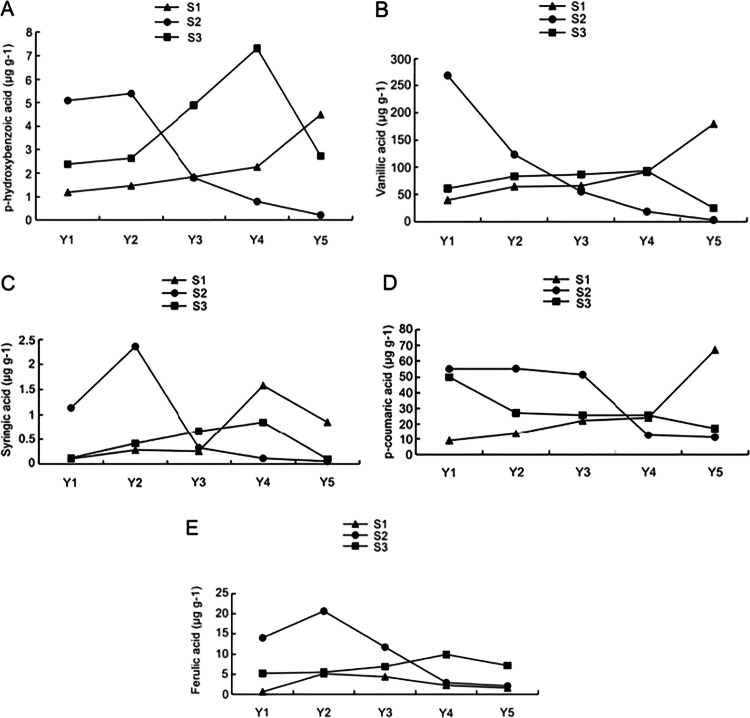
Changes in soil *p*-hydroxybenzoic acid (A), vanillic acid (B), syringic acid (C), *p*-coumaric acid (D), and ferulic acid (E) contents of *Fritillaria taipaiensis* after three fertilizations and cultivation years. S1, chemical fertilizer application; S2, organic fertilizer application; S3, mixed application of chemical and organic fertilizers.

Analysis of phenolic acid contents in the rhizospheric soil of *F*. *taipaiensis* under different fertilization regimes revealed clear trends over the cultivation years (Figure 2A–E). In S3 and S2, the *p*-hydroxybenzoic acid level increased initially and then decreased, peaking in the 4-y-old and 2-y-old soils, respectively (Figure 2A). In contrast, S1 showed a consistent increase with cultivation years. Vanillic acid content in S3 peaked in the 4-y-old soil after an initial increase and subsequent decline; under S1, it increased continuously, while under S2, it decreased (Figure 2B). Syringic acid level under different fertilization treatments showed an initial rise followed by a decline, with peaks in the 4-y soil for S1 and S3, and in the 2-y soil for S2 (Figure 2C). The *p*-coumaric acid level decreased over time under S2 and S3, but increased under S1 (Figure 2D). Ferulic acid level under the three fertilizer applications increased initially and then decreased with cultivation years, with the highest levels in the 2-y soil for S1 and S2, and in the 4-y soil for S3 (Figure 2E). Overall, the phenolic acid level under S1 was significantly higher in the 5-y-old soil than in the 1-y-old soil, indicating an increasing trend over time. Under S2, the highest phenolic acid level occurred in the 1-y-old soil, with the lowest in the 5-y-old soil, showing an overall decline. Under S3, *p*-hydroxybenzoic acid and ferulic acid were slightly higher in the 5-y soil than in the 1-y soil, while the other three phenolic acids were slightly lower.

### Estimation of the accumulation characteristics of phenolic acids

The five phenolic acids exhibited varying degrees of accumulation characteristics in the *F*. *taipaiensis* rhizospheric soils under different fertilizer treatments. To quantitatively analyze these dynamic characteristics, mathematical accumulation models for each phenolic acid were developed. The mean content (Y) of each phenolic acid, measured from rhizospheric soil samples across multiple cultivation years (*t*, the independent variable), was compiled for each fertilizer treatment (S1, S2, and S3) (Table 5). Using SPSS software, a suite of standard mathematical functions was fitted to the dataset for each phenolic acid-treatment combination. The functions tested included linear, conic, exponential, growth, and S curve. The optimal model for each phenolic acid was selected based on *R*^2^ and *P*-value. In S1, the accumulation curve of *p*-hydroxybenzoic acid was best fitted by a conic curve, yielding the highest determination coefficient (*R*² = 0.951, *P* = 0.049). The accumulation curves for vanillic acid and *p*-coumaric acid were best fitted by a growth curve, with *R*² values of 0.914 (*P* = 0.011) and 0.924 (*P* = 0.009), respectively. In S2, the vanillic acid accumulation curve was best fitted by a conic curve, showing the highest *R*² of 0.993 (*P* = 0.009). The *p*-coumaric acid accumulation curve was best fitted with a linear curve (*R*² = 0.799, *P* = 0.041). The accumulation curves for *p*-hydroxybenzoic acid, syringic acid, and ferulic acid were best fitted by a growth curve, with *R*² values of 0.926 (*P* = 0.009), 0.848 (*P* = 0.026), and 0.803 (*P* = 0.040), respectively. In S3, the *p*-coumaric acid accumulation curve was best fitted with an S curve, yielding an *R*² of 0.883 (*P* = 0.018).

**Table 5.** Optimal model of phenolic acid accumulation.

Phenolic Acid	Fertilization	Curve model	Regression equation
*p*-Hydroxybenzoic acid	S1	Conic curve	*Y* = -2.381 + 4.617t − 0.680*t*^2^
	S2	Growth curve	*Y* = 18.234 × exp(−0.827*t*)
Vanillic acid	S1	Growth curve	*Y* = 28.140 × exp(0.337*t*)
	S2	Conic curve	*Y* = 429.827 − 188.106*t* − 20.758*t*^2^
Syringic acid	S1	Exponential curve	*Y* = 0.100*t*1.459
	S2	Growth curve	*Y* = exp(1.670 − 0.899*t*)
*p*-Coumaric acid	S1	Growth curve	*Y* = exp(1.667 + 0.462*t*)
	S2	Linear curve	*Y* = 76.337 − 13.079*t*
	S3	S curve	*Y* = exp(2.794 + 1.118/*t*)
Ferulic acid	S2	Growth curve	*Y* = exp(3.720 − 0.579*t*)

Note: S1, chemical fertilizer application; S2, organic fertilizer application; S3, mixed application of chemical and organic fertilizer.

 The *p*-hydroxybenzoic acid accumulated following a conic curve trend in S1 and a growth curve trend in S2 (Table 5); the vanillic acid accumulated following a growth curve trend in S1 and a conic curve trend in S2; the syringic acid accumulated following an exponential curve trend in S1 and a growth curve trend in S2; the *p*-coumaric acid accumulated following a growth curve, linear curve, and S curve trends in S1, S2, and S3, respectively; the ferulic acid accumulated following a growth curve trend in S2 (Table 5). In S2, all five phenolic acids accumulated following a specific curve trend with the increase in cultivation years; except for ferulic acid, the other four phenolic acids accumulated following specific curve trends with the increase in cultivation years in S1; except for *p*-coumaric acid, the other four phenolic acids did not accumulate following any curve trend with the increase in cultivation years.

### Correlation between phenolic acids

Correlation analysis revealed varying degrees of positive associations among phenolic acid concentrations under different fertilization regimes and cultivation durations (Table 6). Vanillic acid showed positive correlations with *p*-hydroxybenzoic acid (*r* = 0.649, *P* < 0.01) and *p*-coumaric acid (*r* = 0.710, *P* < 0.01). *p*-Hydroxybenzoic acid exhibited significant positive correlations with syringic acid (*r* = 0.614, *P* < 0.05) and ferulic acid (*r* = 0.594, *P* < 0.05). Syringic acid was significantly correlated with vanillic acid (*r* = 0.583, *P* < 0.05) and ferulic acid (*r* = 0.631, *P* < 0.05). These findings suggested interdependent relationships among phenolic acids in *F. taipaiensis* rhizospheric soils, implying potential synergistic interactions under varying fertilization and cultivation conditions.

**Table 6.** Correlation analysis of phenolic acid contents in rhizosphere soil of *Fritillaria taipaiensis.*

Phenolic acid	*p*-Hydroxybenzoic acid	Vanillic acid	Syringic acid	*p*-Coumaric acid
Vanillic acid	0.649**			
Syringic acid	0.614*	0.583*		
*p*-Coumaric acid	0.510	0.710**	0.500	
Ferulic acid	0.594*	0.437	0.631*	0.519*

*P < 0.05; **P < 0.01.

### Correlation between phenolic acids and soil nutrient levels

The concentrations of the five phenolic acids did not exhibit any significant correlation with soil nutrient levels (Table 7). The concentrations of vanillic acid (*r* = 0.685, *P* < 0.01) and ferulic acid (*r* = 0.517, *P* < 0.05) were found to be significantly positively correlated with the organic matter content, respectively. The other three phenolic acids also showed no significant positive correlations with the organic matter content. Additionally, there was no significant correlation observed between pH and the concentrations of the five phenolic acids.

**Table 7.** Correlation analysis of phenolic acids and chemical factors in the rhizospheric soil of *F. taipaiensis.*

Phenolic acids	Available N	Olsen-P	Available K	Organic matter	pH
*p*-Hydroxybenzoic acid	0.476	−0.209	0.068	0.316	0.196
Vanillic acid	0.106	−0.279	0.011	0.685**	−0.121
Syringic acid	0.124	−0.321	−0.137	0.221	−0.031
*p*-coumaric acid	0.114	−0.136	0.286	0.375	0.041
Ferulic acid	0.338	−0.251	0.255	0.517*	0.317

## Discussion

Autotoxicity is driven mainly by the release of secondary metabolites from plants, such as aromatic acids, phenolic acids, and terpenoids, which can lead to soil sickness and affect plant growth, and various environmental and anthropogenic factors also play significant roles.^16^^,^^17^ Phenolic acids are secondary metabolites produced by plants during growth and are the most typical compounds responsible for plant allelopathy. Under continuous cropping and long-term monoculture, the phenolic acids produced by plants can adversely affect plant growth and development. Additionally, when released into the soil, these compounds can promote the spread of plant pathogens and pests.^18^ In this study, all five common phenolic acids were detected in the rhizospheric soil of *F*. *taipaiensis* across different fertilizer treatments and cultivation periods. *p*-Hydroxybenzoic acid can lead to the accumulation of peroxides in maize roots, thereby inhibiting maize growth.^19^ Furthermore, *p*-hydroxybenzoic acid can disrupt the membrane structure of plant cells, making it easier for diseases to infect plant tissues.^20^ Vanillic acid has been shown to effectively inhibit algal growth and exhibits significant toxicity to many plants.^21^ Vanillic acid treatment can significantly reduce the germination rate of plant seeds, alter the composition of the soil microbial community, decrease the population of beneficial microorganisms, and lead to disease accumulation.^22^ *p*-Coumaric acid can notably inhibit the radicle growth of the medicinal plant *Panax quinquefolium* and reduce its photosynthetic efficiency.^23^ Syringic acid and ferulic acid have also been proven to inhibit the seed germination and growth of numerous plants.^24-26^

In this study, the levels of most phenolic acids in the rhizospheric soil of *F*. *taipaiensis* treated with chemical fertilizer (S1) and a combination of chemical and organic fertilizers (S3) exhibited an increasing trend during 1–4 y of cultivation, with the highest concentrations observed in the soil after 4 y. This finding may partially explain the issue of germplasm degradation commonly observed in *F*. *taipaiensis* during the third to fourth years of cultivation.^3^^,^^5^^,^^6^ In fact, the application of organic fertilizer enhances the population of beneficial soil microorganisms, improves the soil microbial community structure, and accelerates the decomposition of phenolic acid allelochemicals, thereby helping to alleviate continuous cropping obstacles in cultivated crops.^27^^,^^28^ Consequently, in this study, although the phenolic acid content in the *F*. *taipaiensis* rhizospheric soil with S2 was higher than that in soils with S1 and S3 in the first year of cultivation, the phenolic acid content in S2 decreased with increasing cultivation years. By the fifth year, the phenolic acid content in S2 was lower than that in S1 and S3. Meanwhile, the phenolic acid content in the rhizospheric soil with S1 showed a continuous upward trend, while the content in the soil with S3 increased initially and then decreased. By the fifth year, the phenolic acid content in S3 was significantly lower than that in S1.

 There exists a certain relationship of promotion and antagonism among the various phenolic acids in the soil.^11^ In this study, a general positive correlation was observed among the different phenolic acids in the rhizospheric soils across various fertilizer treatments, which aligns with previous research on tobacco.^11^^,^^27^ This suggests a synergistic effect between different phenolic acids in the rhizospheric soil of *F*. *taipaiensis*. However, it is worth noting that some studies have also reported antagonistic effects between different phenolic acids in the soil,​​​​​^25^ whereas no negative correlation was found among the phenolic acids in the rhizospheric soil of *F*. *taipaiensis* in this study. Research on the interactions between various phenolic acids in the soil requires further investigation.

 The levels of available N, Olsen-P, available K, and organic matter in the soil are crucial indicators of soil fertility. In this study, the concentrations of available N, Olsen-P, and available K in the rhizospheric soils of *F*. *taipaiensis* treated with S1 and S3 were higher than that in the rhizospheric soil treated with S2, while the organic matter content was lower than that in S2. This finding is consistent with previous studies on various medicinal plants, vegetables, and food crops.^29^^,^^30^ Although the application of organic fertilizer is not as effective as chemical fertilizer in enhancing the content of readily available nutrients in the soil, it can significantly improve the soil structure and yield and quality of crops in the long term.^29^^,^^30^ Although there was no significant correlation between soil pH and most phenolic acid levels in this study, some studies have pointed out that the optimal soil pH value for the growth of *Fritillaria* spp. is approximately 6.66.^31^ The soil pH value with S1 was 5.578, which is considerably lower than the optimal pH for *Fritillaria* spp. growth. In contrast, the pH value with S2 and S3 was closer to the appropriate range for *F*. *taipaiensis* growth. Therefore, appropriate fertilization can enhance the soil nutrient status and structure, thereby improving the growth environment and quality of *F*. *taipaiensis*.

 Previous studies have confirmed that the concentrations of most phenolic acids in the soil exhibit a specific trend of accumulation as the number of continuous cropping years' increases, and models for the accumulation trends of some common soil phenolic acids have been developed through curve estimation.^11^ This study estimated the accumulation trends of five common phenolic acids in the rhizospheric soil of *F*. *taipaiensis* and found that the accumulation patterns of the same phenolic acid varied under different fertilizer treatments. This finding suggests that the accumulation trends of phenolic acids can be influenced by different planting conditions and environmental factors. Therefore, investigating the changing trends and patterns of different phenolic acids with the increase in continuous cropping is highly important. This investigation, when combined with the study of environmental factors and soil microbial populations, will be crucial for understanding the mechanism of action of phenolic acids in continuous cropping obstacles in future research.

## Conclusions

The phenolic acid content in the rhizospheric soil of *F*. *taipaiensis* under different fertilization regimes exhibited a general trend of an initial increase followed by a decrease with the progression of cultivation years, peaking at 4 y of cultivation. As a result, soil phenolic acid profiling could serve as a key biological indicator for soil health monitoring. Crucially, the phenolic acid content in organically fertilized soils became significantly lower than that in chemically fertilized soils over time. This finding indicates that the organic fertilizer application can effectively decelerate the phenolic acid accumulation and reduce the overall pool of phenolic acids, which provides a mechanistic basis for using soil amendments as a tool to manage rhizosphere ecology and improve crop performance.​​​​​​^32^^,^^33^ Additionally, there is generally a synergistic effect among different phenolic acids. The same phenolic acid exhibited varying accumulation trends in the rhizospheric soil of *F*. *taipaiensis* under different fertilizer treatments. Therefore, integrating phenolic acid diagnostics into decision-support systems in precision agriculture could enable farmers to manage soil health precisely, counteract allelopathic autotoxicity, and ultimately promote the long-term viability of medicinal plant cultivations like *F*. *taipaiensis*.

## Institutional review board statement

Not applicable.

## Data Availability

The original contributions presented in the study are included in the article/supplementary material, further inquiries can be directed to the corresponding author.
